# Supplementary material for: Effects of Supplemental Lighting on Flavonoid and Anthocyanin Biosynthesis in Strawberry Flesh Revealed via Metabolome and Transcriptome Co-Analysis
Source: Plants (Basel). 2024 Apr 10;13(8):1070. doi: 10.3390/plants13081070 (PMC11055167; doi:10.3390/plants13081070)
Supplement: Supplementary file 1 [file plants-13-01070-s001.zip › Table S3.pdf]

Table 3 Transcriptome sequencing results of mature strawberries under different light conditions.

Sample	Total reads	Total mapped	Total CDS	Intron	Express gene	Q30
WT1	22759754	80.55%	49878025	1067422	19493	95.62%
WT2	26826631	80.56%	59114737	1285115	19824	96.18%
WT3	24427773	80.28%	53081069	1153718	19734	96.17%
RB1	20759983	79.23%	44480903	1079652	20539	95.05%
RB2	23104510	77.72%	48899025	981864	19954	91.82%
RB3	32915788	78.19%	69771822	1543474	20040	94.05%
UVB1	21232950	79.04%	45500990	965316	20051	94.44%
UVB2	30400897	79.11%	65504670	1393565	19853	94.24%
UVB3	22409493	78.76%	48260158	1037198	19823	93.89%
