# Supplementary material for: Effects of Supplemental Lighting on Flavonoid and Anthocyanin Biosynthesis in Strawberry Flesh Revealed via Metabolome and Transcriptome Co-Analysis
Source: Plants (Basel). 2024 Apr 10;13(8):1070. doi: 10.3390/plants13081070 (PMC11055167; doi:10.3390/plants13081070)
Supplement: Supplementary file 1 [file plants-13-01070-s001.zip › Table S6.pdf]

Table S6 Differential expression of flavonoid biosynthetic and regulatory genes in the strawberry fruits under RB and UVB irradiation.

Gene ID	CK-FR-1	CK-FR-2	CK-FR-3	RB71-FR-1	RB71-FR-2	RB71-FR-3	UV-B-FR-1	UV-B-FR-2	UV-B-FR-3	Types
FvH4_6g16060	159.63	126.80	125.22	205.98	162.89	184.26	137.00	164.64	149.43	PAL
FvH4_7g19130	292.49	244.70	234.62	262.97	250.30	250.60	244.16	261.60	365.14	
FvH4_3g40570	29.00	17.85	26.12	77.05	31.35	101.65	68.39	31.43	76.82	C4H
FvH4_3g30980	11.90	9.26	12.50	16.62	11.47	13.87	14.07	11.52	13.43	
FvH4_6g16460	550.72	596.32	559.70	417.88	546.72	366.18	408.03	552.12	609.05	
FvH4_4g09340	15.55	17.76	18.17	47.70	29.16	31.26	33.82	19.39	29.25	
FvH4_5g07750	6.87	8.64	7.01	13.86	6.39	9.72	12.61	5.20	6.56	4CL
FvH4_6g27940	0.86	1.10	0.63	4.08	3.68	0.44	2.43	0.19	5.52	
FvH4_2g37640	3.49	3.26	2.85	12.64	3.50	10.17	12.88	3.36	4.00	
FvH4_7g33990	13.09	9.43	13.38	19.93	13.82	16.58	14.97	16.42	10.23	
FvH4_7g01160	948.68	1023.81	898.07	1133.45	1159.30	1544.96	976.64	1179.74	1028.29	CHS
FvH4_7g20870	81.06	101.01	77.69	107.06	120.96	139.80	85.77	135.68	74.37	CHI
FvH4_7g25890	1051.37	842.57	947.73	1168.04	1096.88	1110.48	938.26	1083.11	1074.34	
FvH4_1g11810	782.50	610.96	599.47	700.34	671.26	854.19	592.19	800.56	711.74	F3H
FvH4_3g02980	6.25	7.94	4.71	17.83	11.13	5.02	5.12	6.04	4.75	
FvH4_2g39520	353.98	417.38	280.65	538.50	516.73	485.52	384.00	484.04	408.22	DFR
FvH4_2g39530	543.99	432.82	504.63	959.55	608.33	818.65	612.31	686.16	484.34	
FvH4_5g20430	18.85	29.33	31.04	9.53	16.01	3.88	6.54	15.92	11.83	FLS
FvH4_7g24550	30.31	14.76	23.55	22.02	13.39	19.19	39.74	24.13	21.69	
FvH4_5g38050	12.38	24.87	29.24	59.57	16.28	13.01	35.51	17.98	10.86	
FvH4_1g02120	644.10	463.92	536.36	228.51	467.41	297.88	264.40	488.08	527.91	ANS
FvH4_5g01170	1009.61	923.43	786.79	1171.34	1052.43	1069.33	971.43	1111.53	1208.21	
FvH4_6g34910	2.59	2.54	3.23	3.58	3.43	4.68	3.73	3.04	3.87	F3'H
FvH4_6g34950	5.54	6.02	5.43	11.09	4.38	3.97	12.41	2.58	4.96	F3'5'H
FvH4_5g10440	8.60	6.92	8.17	9.88	9.23	10.62	9.74	9.95	8.37	OMT
FvH4_1g12660	56.31	39.32	33.91	33.68	33.01	21.58	36.79	39.64	55.68	
FvH4_1g19400	12.60	11.22	8.89	7.22	9.17	4.71	7.04	8.47	13.74	
FvH4_1g19390	34.15	16.48	16.70	9.20	16.22	10.95	17.28	24.04	29.13	
FvH4_5g13300	2.96	4.09	3.81	10.95	4.61	8.07	9.09	4.49	3.97	
FvH4_6g50530	21.86	19.77	30.37	20.08	20.98	16.67	13.07	20.53	15.81	
FvH4_3g10810	8.28	18.91	8.87	8.55	6.16	12.36	9.47	8.14	10.79	
FvH4_4g11780	18.63	32.98	17.87	9.58	9.76	8.81	10.24	6.61	19.73	UFGT
FvH4_7g16110	21.17	14.70	12.02	67.81	19.58	43.74	55.64	12.74	29.45	
FvH4_6g39410	35.78	70.17	48.88	38.43	30.52	56.38	30.81	37.48	45.53	
FvH4_6g39380	13.28	14.21	15.85	28.17	16.00	14.09	13.52	8.23	34.98	
FvH4_1g13990	25.68	24.25	31.81	60.47	41.18	54.43	46.52	37.49	26.68	
FvH4_5g23800	5.29	5.66	6.37	11.59	7.16	5.91	9.07	5.51	7.75	
