# Supplementary material for: Effects of Supplemental Lighting on Flavonoid and Anthocyanin Biosynthesis in Strawberry Flesh Revealed via Metabolome and Transcriptome Co-Analysis
Source: Plants (Basel). 2024 Apr 10;13(8):1070. doi: 10.3390/plants13081070 (PMC11055167; doi:10.3390/plants13081070)
Supplement: Supplementary file 1 [file plants-13-01070-s001.zip › Table S7.pdf]

Gene	TF	Gene	TF
FvH4_1g21210	AP2/ERF	FvH4_3g03530	LFY
FvH4_4g28030	AP2/ERF	FvH4_7g20130	LOB
FvH4_6g07580	ARID	FvH4_4g27090	MYB
FvH4_6g31930	AUX/IAA	FvH4_7g23990	MYB
FvH4_2g22520	AUX/IAA	FvH4_5g06070	MYB
FvH4_2g38760	B3-ARF	FvH4_7g00180	MYB
FvH4_2g19790	bHLH	FvH4_6g43450	MYB
FvH4_7g24720	bHLH	FvH4_1g13110	MYB
FvH4_4g27100	bHLH	FvH4_5g21670	NAC
FvH4_7g00670	bHLH	FvH4_2g16180	NAC
FvH4_2g36400	bZIP	FvH4_4g31070	NAC
FvH4_4g27390	C2C2-Dof	FvH4_7g18010	NAC
FvH4_3g33660	C2C2-Dof	FvH4_1g25150	NAC
FvH4_3g04730	C2C2-Dof	FvH4_6g15670	NAC
FvH4_3g35790	C2C2-Dof	FvH4_7g18000	NAC
FvH4_5g13390	C2C2-Dof	FvH4_2g27430	NAC
FvH4_c6g00070	C2H2	FvH4_6g20510	NF-YA
FvH4_2g22950	C2H2	FvH4_1g08160	NF-YA
FvH4_2g26420	CSD	FvH4_6g34360	NF-YA
FvH4_2g29840	DBP	FvH4_3g29360	NF-YB
FvH4_1g04080	GRAS	FvH4_7g23550	Others
FvH4_2g32670	GRF	FvH4_2g19810	Others
FvH4_1g08440	GRF	FvH4_1g11530	Others
FvH4_2g39270	HB-BELL	FvH4_7g11220	Others
FvH4_5g15520	HB-HD-ZIP	FvH4_4g31520	Others

FvH4_6g50570	HB-HD-ZIP	FvH4_2g13840	Trihelix
FvH4_7g17320	HB-HD-ZIP	FvH4_2g13850	Trihelix
FvH4_7g24360	HMG	FvH4_5g39060	WRKY
FvH4_1g16030	HSF	FvH4_3g06200	WRKY
FvH4_6g17890	HSF		

Table S7 Transcription factors in mature strawberries under different lighting conditions.
