# Supplementary material for: Effects of Supplemental Lighting on Flavonoid and Anthocyanin Biosynthesis in Strawberry Flesh Revealed via Metabolome and Transcriptome Co-Analysis
Source: Plants (Basel). 2024 Apr 10;13(8):1070. doi: 10.3390/plants13081070 (PMC11055167; doi:10.3390/plants13081070)
Supplement: Supplementary file 1 [file plants-13-01070-s001.zip › Table S8.pdf]

Table S8 Co-expression network of transcription factors and structural genes with DEM under UV-B light treatment.

Gene	Metabolite
FvH4_3g40570	Kaempferol-6,8-di-C-glucoside-7-O-glucoside
FvH4_3g40570	Vitexin-2''-O-galactoside
FvH4_3g40570	Kaempferol-3-O-(2''-O-acetyl)glucoside
FvH4_3g40570	Apigenin-7-O-Gentiobioside
FvH4_3g40570	Quercetin-3-O-(6''-O-acetyl)glucoside
FvH4_3g40570	Kaempferide-3-O-(6''-malonyl)glucoside
FvH4_3g40570	Liquiritigenin-4'-O-Glucoside (Liquiritin)
FvH4_3g40570	Isorhamnetin-3-O-(6''-malonyl)glucoside
FvH4_3g40570	Naringenin-4'-O-glucosid
FvH4_3g40570	Luteolin-3'-O-glucoside
FvH4_3g40570	Phloretin-4'-O-(6''-p-Coumaroyl)glucoside
FvH4_3g40570	Kaempferol-3-O-(6''-O-acetyl)glucoside
FvH4_3g40570	Luteolin-7-O-(6''-malonyl)glucoside
FvH4_3g40570	Tricin-4'-O-syringic acid
FvH4_3g40570	Kaempferol-3-O-(6''-malonyl)galactoside
FvH4_3g40570	Quercetin-3-O-(6''-O-acetyl)galactoside
FvH4_3g40570	Vitexin-2''-O-rhamnoside
FvH4_3g40570	Naringenin-7-O-(6''-malonyl)glucoside
FvH4_3g40570	Kaempferol-3-O-(6''-malonyl)glucoside
FvH4_3g40570	Kaempferol-3,7-O-diglucoside
FvH4_3g40570	Kaempferol-3-O-rutinoside(Nicotiflorin)
FvH4_3g40570	C-glucosyl-C-arabinosyl-2-hydroxynaringen

FvH4_3g40570	Kaempferol-7-O-glucoside
FvH4_3g40570	Gallocatechin-(4α→8)-gallocatechin
FvH4_3g40570	Eriodictyol-8-C-glucoside-4'-O-glucosid
FvH4_3g40570	MethylChrysoeriol-8-C-glucoside
FvH4_3g40570	Peonidin-3-O-glucoside
FvH4_3g40570	Kaempferol-3-O-galactoside (Trifolin
FvH4_4g09340	Kaempferol-6,8-di-C-glucoside-7-O-glucoside
FvH4_4g09340	Vitexin-2"-O-galactoside
FvH4_4g09340	Kaempferol-3-O-(2"-O-acetyl)glucoside
FvH4_4g09340	Apigenin-7-O-Gentiobioside
FvH4_4g09340	Quercetin-3-O-(6"-O-acetyl)glucoside
FvH4_4g09340	Kaempferide-3-O-(6"-malonyl)glucoside
FvH4_4g09340	Liquiritigenin-4'-O-Glucoside (Liquiritin)
FvH4_4g09340	Isorhamnetin-3-O-(6"-malonyl)glucoside
FvH4_4g09340	Naringenin-4'-O-glucosid
FvH4_4g09340	Luteolin-3'-O-glucoside
FvH4_4g09340	Phloretin-4'-O-(6"-p-Coumaroyl)glucoside
FvH4_4g09340	Kaempferol-3-O-(6"-O-acetyl)glucoside
FvH4_4g09340	Luteolin-7-O-(6"-malonyl)glucoside
FvH4_4g09340	Tricin-4'-O-syringic acid
FvH4_4g09340	Kaempferol-3-O-(6"-malonyl)galactoside
FvH4_4g09340	Quercetin-3-O-(6"-O-acetyl)galactoside
FvH4_4g09340	Vitexin-2"-O-rhamnoside
FvH4_4g09340	Naringenin-7-O-(6"-malonyl)glucoside
FvH4_4g09340	Kaempferol-3-O-(6"-malonyl)glucoside
FvH4_4g09340	Kaempferol-3,7-O-diglucoside

FvH4_4g09340	Kaempferol-3-O-rutinoside(Nicotiflorin)
FvH4_4g09340	C-glucosyl-C-arabinosyl-2-hydroxynaringen
FvH4_4g09340	Kaempferol-7-O-glucoside
FvH4_4g09340	Gallocatechin-(4α→8)-gallocatechin
FvH4_4g09340	Eriodictyol-8-C-glucoside-4'-O-glucosid
FvH4_4g09340	MethylChrysoeriol-8-C-glucoside
FvH4_4g09340	Peonidin-3-O-glucoside
FvH4_4g09340	Kaempferol-3-O-galactoside (Trifolin
FvH4_1g21210	Phloretin-4'-O-(6''-p-Coumaroyl)glucoside、
FvH4_1g21210	Quercetin-3-O-(2'''-O-p-coumaroyl)sophoroside-7-O-glucoside
FvH4_1g21210	Vitexin-2''-O-galactoside
FvH4_1g21210	Apigenin-7-O-Gentiobioside
FvH4_1g21210	ricin-4'-O-syringic acid
FvH4_1g21210	Eriodictyol-8-C-glucoside-4'-O-glucoside
FvH4_1g21210	Vitexin-2''-O-rhamnoside
FvH4_1g21210	C-glucosyl-C-arabinosyl-2-hydroxynaringenin
FvH4_2g22520	Phloretin-4'-O-(6''-p-Coumaroyl)glucoside、
FvH4_2g22520	Quercetin-3-O-(2'''-O-p-coumaroyl)sophoroside-7-O-glucoside
FvH4_2g22520	Vitexin-2''-O-galactoside
FvH4_2g22520	Apigenin-7-O-Gentiobioside
FvH4_2g22520	ricin-4'-O-syringic acid
FvH4_2g22520	Eriodictyol-8-C-glucoside-4'-O-glucoside
FvH4_2g22520	Vitexin-2''-O-rhamnoside
FvH4_2g22520	C-glucosyl-C-arabinosyl-2-hydroxynaringenin
FvH4_2g22520	Kaempferol-7-O-glucoside
FvH4_2g22520	Kaempferol-6,8-di-C-glucoside-7-O-glucoside

FvH4_2g22520	Quercetin-3-O-(6"-O-acetyl)galactoside
FvH4_2g22520	Naringenin-7-O-(6"-malonyl)glucoside
FvH4_2g22520	Luteolin-7-O-(6"-malonyl)glucoside
FvH4_2g22520	Kaempferol-3-O-(6"-malonyl)galactoside
